# Chronic disease prevention literacy and its influence on behavior and lifestyle: a cross-sectional study in Xinjiang, China

**DOI:** 10.1186/s12889-023-16884-1

**Published:** 2023-10-11

**Authors:** Alimire Abudireyimu, Yinxia Su, Conghui Hu, Yuanyuan Li, Hua Yao

**Affiliations:** 1 School of Public Health, Xinjiang Medical University, Urumqi, Xinjiang China; 2 School of Medical Engineering and Technology, Xinjiang Medical University, Urumqi, Xinjiang China; 3 First Affiliated Hospital of Xinjiang Medical University, Urumqi, Xinjiang China

**Keywords:** Kyrgyz, Chronic diseases, Literacy, Behavioral lifestyle

## Abstract

**Objective:**

To understand the status and influencing factors of Kyrgyz chronic disease prevention literacy, and to explore the impact of chronic disease prevention literacy on behavior and living habits.

**Method:**

Using stratified sampling method, Kyrgyz residents aged ≥ 18 years in Artush City, Aheqi County and Ucha County were surveyed by questionnaire.

**Results:**

A total of 10,468 subjects were investigated, and the literacy rate of chronic disease prevention in Kyrgyz was 11.2%. The results of Logistic regression analysis showed that the literacy rate of chronic disease prevention was low among people with low education level, herdsmen, low income, urban and chronic disease (*P* < 0.05). Residents with chronic disease prevention literacy were more inclined to not smoke, not drink alcohol, drink milk every day, eat soy products every month, eat whole grains every day (*P* < 0.05).

**Conclusion:**

The literacy level of chronic disease prevention of Kyrgyz residents in Kezhou has been improved, but it is still at a low level compared with another subcategories. The behavioral lifestyle is related to the literacy level of chronic disease prevention. Therefore, local health promotion strategies should be developed to improve the literacy level of chronic disease prevention and promote the formation of good behavioral and living habits.

**Supplementary Information:**

The online version contains supplementary material available at 10.1186/s12889-023-16884-1.

## Introduction

Chronic diseases have become the leading cause of human death [1]. Since most chronic diseases are closely related to patients’ personal concepts and living habits, it is significant for individuals to actively participate and self-manage in the prevention and treatment of chronic diseases [2]. Research shows that improving the chronic disease prevention literacy of the population is an effective measure to improve the efficiency of individual self-management behavior [3]. Chronic disease prevention literacy is one of the main contents in health literacy evaluation system. It refers to people’s basic knowledge, health behaviors and lifestyles, and chronic disease self-management abilities related to the prevention and treatment of common chronic noncommunicable diseases, which should be possessed by healthy individuals in order to maintain and promote health [4]. Research shows that unhealthy behaviors and lifestyles in daily life have adverse effects on health [5], and people with high health literacy are more likely to have healthy lifestyles and eating habits [6]. China is a unified multi-ethnic country, and the Kyrgyz are one of the ethnic minorities in China. According to the seventh National Census, the Kyrgyz population in China in 2020 was 204,402, of which 80.16% of Kyrgyz residents live in Kizilsu Kirghiz Autonomous Prefecture (abbreviated as Kezhou) in southern Xinjiang at an altitude of 1500 ~ 4000 m [7]. The rest are distributed in Xinjiang’s Yili, Aksu, Kashgar and Heilongjiang Provinces. They are mainly engaged in animal husbandry, agriculture and handicraft industry, and their diet and daily life are characterized by a nomadic lifestyle. According to the results of Kyrgyz health literacy survey in Xinjiang, 2018 [8], the health literacy rate of Kyrgyz is 4.76%, which is lower than the overall health literacy level of Western China in the same year (13.23%) [9]. At present, there are few studies on the chronic disease prevention literacy of Kyrgyz residents, and there are no reports on the correlation between living and eating habits and chronic disease prevention literacy. This study aims to further understand the current status of chronic disease prevention literacy among Kyrgyz residents in Kezhou, Xinjiang, and analyze the relationship between living and eating habits and chronic disease prevention literacy.

## Materials and methods

### Population

In this study, the Permanent residents of the Kyrgyz ethnic group in Artush City, Aheqi County and Ucha County in Kezhou Region of Xinjiang from July to December 2021 were selected as the study subjects. Inclusion criteria: Permanent residents aged ≥ 18 years old who lived in Kezhou for more than 6 of the previous 12 months at the time of the survey; Exclusion criteria: those who do not meet the age, those who cannot cooperate with the survey normally, and those who are not residents. This study has been approved by the Ethics Committee of the First Affiliated Hospital of Xinjiang Medical University (20160317-02), and the participants have signed informed consent.

### Sample size

Sampling was carried out using the stratified sampling method. Referring to the 2018 health literacy monitoring results of Chinese residents, the health literacy level of residents in the central and western regions was 13.23%, the 95% confidence interval was taken, and the allowable error was calculated according to 0.02, and the sample size $N={Z}_{\alpha /2}^{2}\times \frac{p\times (1-p)}{{\delta }^{2}}$ was finally obtained as 1103 people. In urban and rural areas, according to different genders and ages, the minimum sample size was calculated N= 1103 people per layer×10 layers = 11,030 people. In the end, a total of 13,000 questionnaires were distributed, 10,754 questionnaires were recovered, and invalid questionnaires were excluded, and 10,468 valid questionnaires were valid, with an effective rate of 97.34%.

### Questionnaire design and measurement

The Questionnaire of this study-Xinjiang Ethnic Minority Areas Health Culture Survey Questionnaire, which is suitable for ethnic minority areas, is designed through literature research method, field research, and reference to the contents of the “National Residents Health Literacy Monitoring Questionnaire (2015 Edition)” [10] by extracting some relevant questions from which, and revised by Delphi method [11]. Then, a preliminary survey was carried out, and the reliability and validity of the questionnaire were analyzed and adjusted. The Cronbach’s *α* coefficient of the questionnaire was 0.834, the validity was 0.821 [8, 12, 13]. Questionnaires are conducted on the spot, investigators are trained uniformly, and at least one translator is provided. If the respondents are unable to complete the questionnaire due to their limited educational level, the translator will explain to the respondents and assist them in answering the questionnaire, but cannot suggest the answer.

The questions of health literacy were divided into six subcategories: scientific view of health, health information literacy, infectious disease prevention literacy, chronic disease prevention literacy, safety and first aid literacy and basic medical literacy. The questionnaire consisted of 68 questions (43 single-answer and 25 multiple-answer), with a full score of 93: single-answer (1 point for single-answers consistent with standard answers, 0 point for errors); multiple-answer (if the multiple-answer is exactly the same as the standard answer, the score will be 2 points, and if the wrong answer is missed, the score will be 0 points), no-response questions were counted by 0 points. A respondent is defined as having adequate health literacy if their total score is at least 80% of the full score.

Content related to chronic disease prevention literacy serves as a data source for this study. Among them, there are 7 questions in chronic disease prevention literacy (Please see Attachment for the original items), which are divided into 2 single-answer and 5 multiple- answer, with a total of 12 points. If the questionnaire score is more than 80% of the total score, that is, the total score ≥ 9 points, it is deemed that the respondent has the literacy of chronic disease prevention, otherwise it is regarded as not having the literacy of chronic disease prevention.

Our study also collected basic information on the subjects` demographic characteristics, lifestyle and eating habits and health status. We retrieved information on age, gender, education, occupation, marital status, household income etc. Health status-related content includes whether suffering from chronic diseases, as well as the type of disease, the number of years of illness, and the evaluation of self-health status.

### Statistical analyses

Establish a database and double entry through Epidata3.1, and use SPSS23.0 for statistical analysis. Descriptive analysis of the basic situation of residents, t-test, analysis of variance and chi-square test was used for the inter group comparison of measurement data and counting data. First, logistic regression analysis with chronic disease prevention literacy as the dependent variable was used to test the correlation with socio-demographic characteristics. Then, a multivariate logistic regression model was constructed with different behaviors, lifestyles and eating habits as dependent variables, and whether people had chronic disease prevention literacy as independent variables. In the model, the effects of gender, age, education, occupation, marital status, family per capita monthly income, region and family size were controlled. The test level is *α* = 0.05.

## Results

### Characteristics of the participants

Among the 10,468 survey respondents, 54.0% were women, the average age was 47.28 ± 18.97, junior high school education accounted for 39.9%, livestock residents accounted for 26.9%, married 68.9%, rural residents accounted for 78.4%, and the per capita monthly household income was 1000 ~ 1999 yuan and 2000 ~ 4999yuan, accounting for 27.4% and 29.4% respectively, as shown in Table 1.

**Table 1:** Distribution of general situation and chronic disease prevention literacy levels of participants n (%)

Characteristics	All^(p)^	Chronic disease prevention literacy^(r)^		${\varvec{x}}^{2}$	*p*
		adequate	inadequate		
Gender (%)				0.453	0.501
Male	4813(46.0)	552(11.5)	4261(88.5)		
Female	5655(54.0)	625(11.1)	5030(88.9)		
Age (Year)				7.874	0.020
18 ~ 39	3842(36.7)	447(11.6)	3395(88.4)		
40 ~ 59	2881(27.5)	351(12.2)	2530(87.8)		
60~	3745(35.8)	379(10.1)	3366(89.9)		
Education				24.268	<0.001
Junior High School and below	8592(82.1)	905(10.5)	7678(89.5)		
High school and above	1876(17.9)	272(14.5)	1604(85.5)		
Occupation				47.542	<0.001
Personnel of government agencies, enterprises and institutions	425(4.1)	57(13.4)	368(86.6)		
Private enterprises, business (industry) personnel	1021(9.8)	148(14.5)	873(85.5)		
Professional and technical personnel	913(8.7)	119(13.0)	793(87.0)		
Agroforestry laborer	789(7.5)	93(11.8)	696(88.2)		
Shepherd	2813(26.9)	249(8.9)	2564(91.1)		
Student	1533(14.6)	175(11.4)	1358(88.6)		
Housewife	1148(11.0)	124(10.8)	1024(89.2)		
Retiree	680(6.5)	102(15.0)	578(85.0)		
Unemployed	528(5.0)	59(11.2)	469(88.8)		
Other	618(5.9)	51(8.3)	567(91.7)		
Marital status				0.135	0.935
Single	2489(23.8)	275(11.1)	2214(88.9)		
Married	7217(68.9)	815(11.3)	6402(88.7)		
Divorced/Widowed	762(7.3)	87(11.4)	675(88.6)		
Household income (Yuan/month)				138.187	<0.001
<500	745(7.1)	49(6.6)	696(93.4)		
500 ~ 999	1346(12.9)	112(8.3)	1234(91.7)		
1000 ~ 1999	2862(27.3)	230(8.0)	2632(92.0)		
2000 ~ 4999	3080(29.4)	501(16.3)	2579(83.7)		
5000 ~ 9999	2013(19.2)	245(12.2)	1768(87.8)		
≥ 10,000	422(4.0)	40(9.5)	382(90.5)		
Region				7.548	0.006
Urban	2264(21.6)	218(9.6)	2046(90.4)		
Rural	8204(78.4)	959(11.7)	7245(88.3)		
Family size (Person)				4.576	0.101
1 ~ 3	1599(15.3)	203(12.7)	1396(87.3)		
4 ~ 6	7678(73.3)	851(11.1)	6827(88.9)		
≥ 7	1191(11.4)	123(10.3)	1068(89.7)		

^(p)^percentage; ^(r)^rate of literacy of chronic disease prevention

### Health literacy of six categories of participants

Among these six categories, the literacy rate of chronic disease prevention is the lowest, which is 11.2%, as shown in Table 2.

**Table 2:** Health literacy of six categories of participants

Categories	n	%
Scientific view of health	1269	12.1
Health information literacy	1767	16.9
Infectious disease prevention literacy	1487	14.2
Chronic disease prevention literacy	1177	11.2
Safety and first aid literacy	3117	29.8
Basic medical literacy	2451	23.4

### Analysis on chronic disease prevention literacy and health status of participants

The chi-square test showed that the residents with chronic diseases had a lower rate of chronic disease prevention literacy (7.6%) than the residents without chronic diseases (12.7%). The self-assessed health status with poor (5.7%) and relatively poor (11.4%) had a low rate of chronic disease prevention literacy than the relatively good (14.2%) group, and the difference was statistically significant (*P* < 0.001). In the population with chronic diseases, there was no significant difference in the level of chronic disease prevention and control literacy between the number of chronic diseases and the number of years of illness (*P* > 0.05), as shown in Table 3.

**Table 3:** Correlation analysis between chronic disease prevention literacy and health status of participants n (%)

Variables	All^(p)^	Chronic disease prevention literacy^(r)^		${\varvec{x}}^{2}$	*p*
		Adequate	Inadequate		
Have a chronic disease				55.698	<0.001
No	7382(70.5)	940(12.7)	6442(87.3)		
Yes	3086(29.5)	237(7.6)	2849(92.3)		
Number of chronic diseases				3.684	0.159
One disease	2603(84.4)	190(7.3)	2413(92.7)		
Two diseases	383(12.4)	36(9.4)	347(90.6)		
Three + diseases	100(3.2)	11(11.0)	89(89.0)		
Years of chronic disease/Year				10.451	0.015
<1	894(29.0)	50(5.6)	844(94.4)		
1 ~ 4	1749(56.7)	141(8.1)	1608(91.9)		
5 ~ 9	265(8.6)	28(10.6)	237(89.4)		
≥ 10	178(5.8)	18(10.1)	160(89.9)		
Self-assessed health status				28.873	<0.001
Poor	53(0.5)	3(5.7)	50(94.3)		
Relatively poor	402(3.8)	46(11.4)	356(88.6)		
Fair	1801(17.2)	169(9.4)	1632(90.6)		
Relatively good	2250(21.5)	319(14.2)	1931(85.8)		
Good	5962(57.0)	640(10.7)	5322(89.3)		

^(p)^percentage; ^(r)^rate of literacy of chronic disease prevention

### Analysis of influencing factors of chronic disease prevention literacy of participants

The results of binary logistic regression analysis showed that people with high school and above were 1.26 times (*OR*: 1.264, 95% *CI*: 1.077;1.484) more likely to have chronic disease prevention literacy than junior high school and below; Herders and others were 1.46 times (*OR*: 0.684, 95% *CI*: 0.494;0.944) and 1.80 times (*OR*: 0.557, 95% *CI*: 0.369;0.839) less likely to have chronic disease prevention literacy than those working in government agencies, respectively; The per capita monthly household income with 2000–4999 yuan and with 5000–9999 yuan were 2.48 times (*OR*: 2.485, 95% *CI*: 1.822;3.388) and 1.77 times (*OR*: 1.773, 95% *CI*: 1.729;2.457) more likely to have chronic disease prevention literacy than the average household monthly income of < 500 yuan; Family size of 4–6 and ≥ 7 persons were 1.35 times (OR:0.741, 95%CI:0.622 ~ 0.883) and 1.43 times (OR:0.698, 95%CI:0.543 ~ 0.897) less likely to have chronic disease prevention literacy than that in a family size of 1–3 persons, respectively; Rural populations were 1.39 times (*OR*: 1.394, 95% *CI*: 1.182;1.645) more likely to have chronic disease prevention literacy than urban population; People without chronic diseases were 1.71 times (*OR*: 1.709, 95%*CI*: 1.450;2.013) more likely to have chronic disease prevention literacy than those with chronic diseases, see Table 4.

**Table 4:** Multivariate logistic regression analysis of chronic disease prevention literacy level of participants

Characteristics	*P*	*OR*	(95%*CI*)	
			Upper	Lower
Education				
Junior High School and below		1		
High school and above	0.004	1.264	1.077	1.484
Occupation				
Personnel of government agencies, enterprises and institutions		1		
Private enterprises, business (industry) personnel	0.876	1.028	0.729	1.450
Professional and technical personnel	0.491	0.884	0.623	1.255
Agroforestry laborer	0.271	0.815	0.566	1.173
herder	0.021	0.684	0.495	0.944
Student	0.438	0.847	0.556	1.290
Housewife	0.072	0.723	0.507	1.030
Retiree	0.287	1.221	0.845	1.765
Unemployed	0.235	0.763	0.488	1.192
Other	0.005	0.557	0.369	0.839
Household income (Yuan/month)				
<500		1		
500 ~ 999	0.180	1.271	0.895	1.806
1000 ~ 1999	0.307	1.183	0.857	1.632
2000 ~ 4999	<0.001	2.485	1.822	3.388
5000 ~ 9999	0.001	1.773	1.279	2.457
≥ 10,000	0.176	1.356	0.872	2.109
Family size (Person)				
1 ~ 3		1		
4 ~ 6	0.001	0.741	0.622	0.883
≥ 7	0.005	0.698	0.543	0.897
Region				
Urban		1		
Rural	<0.001	1.394	1.182	1.645
Have a chronic disease				
Yes		1.000		
No	<0.001	1.709	1.450	2.013

### The impact of chronic disease prevention literacy on behavior and lifestyle of participants

Among the survey respondents, most people slept ≥ 7 h a day, accounting for 52.6%; smokers account for 20.7%, non-smokers account for 47.1%; drinkers account for 25.2%, non-drinkers account for 74.8%; 78.1% of the population exercised for more than 3 times a week; The population with a light diet accounted for 38.8%, see Table 5. Taking different behaviors and lifestyles as dependent variables, and having chronic disease prevention literacy as independent variables, a logistic regression model was constructed. After controlling for gender, age, educational level, occupation, marital status, monthly household income, region and family size in the model, the results showed that people with having chronic disease prevention literacy were more inclined to not smoke, not drink alcohol, drink milk every day, eat soy products every month, eat whole grains every day (*P* < 0.05), see Table 6.

**Table 5:** Behavior and lifestyle of participants

Variable	N	Percentage (%)
Daily sleep time/h		
<7	4957	47.4
≥ 7	5511	52.6
Smoking status		
Smoking	2163	20.7
Have quit smoking	3373	32.2
Not smoking	4932	47.1
Drinking status		
Drinking	2643	25.2
Not Drinking	7825	74.8
Exercise for more than 3 times a week		
Yes	8176	78.1
No	2292	21.9
Diet taste		
Salty	2313	22.1
Sweet	3053	29.2
Oily	1042	10.0
Mild	4060	38.8
Eat vegetables and fruits every day		
Yes	7865	75.1
No	2603	24.9
Drink milk (cow, horse, camel) every day		
Yes	7241	69.2
No	3227	30.8
Eating soy products monthly		
Yes	4571	43.7
No	5897	56.3
Eating whole grains every day		
Yes	4170	39.8
No	6298	60.2
Meal pattern		
Irregular	402	3.8
Eat multiple meals	949	9.1
Eat smaller meals	1305	12.5
Three meals a day	7608	72.7
Other	204	1.9

**Table 6:** Effects of chronic disease prevention literacy on behavior and lifestyle of participants

Variables	Model 1^a^			Model 2^b^		
	*P*	*OR*	*95%CI*	*P*	*OR*	*95%CI*
Sleeping time ≥ 7 h/d	0.787	1.017	(0.901 ~ 1.148)	0.301	0.935	(0.824 ~ 1.062)
Not smoking	0.001	1.501	(1.266 ~ 1.780)	<0.001	1.370	(1.144 ~ 1.640)
Not Drinking	<0.001	1.720	(1.468 ~ 2.015)	<0.001	1.667	(1.412 ~ 1.969)
Exercise for more than 3 times a week	0.342	1.075	(0.926 ~ 1.248)	0.619	1.039	(0.893 ~ 1.210)
Mild diet	0.620	0.961	(0.822 ~ 1.124)	0.389	0.931	(0.792 ~ 1.095)
Eat vegetables and fruits every day	0.190	0.912	(0.794 ~ 1.047)	0.118	0.894	(0.776 ~ 1.029)
Drink milk every day	<0.001	1.345	(1.171 ~ 1.544)	<0.001	1.292	(1.124 ~ 1.487)
Eating whole grains every day	<0.001	1.312	(1.161 ~ 1.482)	<0.001	1.337	(1.181 ~ 1.513)
Eating soy products monthly	<0.001	1.742	(1.542 ~ 1.969)	<0.001	1.666	(1.472 ~ 1.886)
Three meals a day	0.031	1.483	(1.037 ~ 2.122)	0.084	1.376	(0.958 ~ 1.976)

^a^ not adjusted, ^b^ adjusted gender, age, educational level, occupation, marital status, family per capita monthly income, region, family size

## Discussion

The results of this study show that the chronic disease prevention literacy level of Kyrgyz residents in Kezhou, Xinjiang was 11.2%, which was higher than the 2018 Kyrgyz chronic disease prevention literacy in Kezhou (7.13%) [8], indicating that the chronic disease prevention literacy level of Kyrgyz residents has improved, but it is still far behind the goal of achieving residents’ health literacy level of 22% by 2022 as required in the “Healthy China Action” [14]. Therefore, it is recommended that in the future health promotion work, health knowledge promotion suitable for the region should be carried out in a targeted manner, the accessibility of health education should be enhanced, and the chronic disease prevention literacy of Kyrgyz residents should be effectively improved.

In the relationship between chronic disease prevention literacy and health status of the Kyrgyz people, it was found that people with chronic diseases have lower chronic disease prevention literacy than those without chronic diseases, and the self-assessed groups of poor and relatively poor health status have a lower rate of chronic disease prevention literacy than those with relatively good health status, which is consistent with the findings of Liu L et al. [15]. This is related to the fact that people with sufficient chronic disease prevention literacy are younger, have higher levels of education, and have higher income levels [16, 17].

Binary logistic regression in our study did not show an association between age and chronic disease prevention literacy. However, other studies have reported that age is an important factor affecting health literacy [18, 19]. This is related to the elderly’s reduced learning, understanding, memory abilities, and their limited ability to discern health information. A lower education level is a negative factor in chronic disease prevention literacy [20, 21]. The level of education determines people’s ability to acquire, understand, and apply health knowledge. A higher education level is more inclined to pursue healthy behaviors and lifestyles. Research Results showed that private enterprises, businesses, and industrial personnel had the highest rate of chronic disease prevention literacy (11.5%), while that of herdsmen had the lowest (4.6%). People working in the private sector and in commercial industries have extensive network, and have many ways to obtain health information. However due to their nomadic life, herdsmen have few learning opportunities, many inherent cognitions and living habits are difficult to change, and the ability to accept and understand new things is insufficient, resulting in low literacy levels of chronic disease prevention. In our chi-square test analysis, there was no correlation between family size and chronic disease prevention literacy, but the logistic regression analysis showed that larger the family size, the lower the rate of chronic disease prevention literacy. This may be related to the large the family size, and the relatively high financial pressure on the family, resulting in insufficient attention to their own health. A number of studies have shown that income level is related to health status [22, 23], which is consistent with the results of this paper that is people with higher income levels have higher chronic disease prevention literacy. Income level will affect residents’ eating and living habits, ability to utilize health resources [24]. Only when our income level meets our basic needs can we afford to invest in wellness.

Interestingly, this study found that rural residents have higher rates of chronic disease prevention literacy than urban residents, which contradicts previous research results [18, 19, 25]. In recent years, with the in-depth advancement of my country’s rural revitalization strategy, the rural economy has developed rapidly, and the poor have been lifted out of poverty in an all-round way. The Internet has been introduced into every household, and there are more channels for receiving health information. Income level has gradually increased. Residents are willing to master more health information skills to care for and maintain their own health [26].

Research shows that chronic disease prevention literacy is closely related to personal health-related behaviors and lifestyles, as well as health status [27]. People with sufficient health literacy are significantly less likely to have high-risk health behaviors, and are more likely to have regular physical examinations, have good health status, and have more access to health information [28]. The results of this study show that the higher the literacy of chronic disease prevention, the more Kyrgyz residents tend to not smoke, not drink alcohol, drink milk every day, eat soy products every month, eat coarse grains every day. Improving the literacy of chronic disease prevention and treatment can promote people to develop good living and eating habits, thereby preventing the occurrence and development of chronic diseases [29]. A study, Wang et al. [30] conducted in China, the Kazakh had a lower prevalence of lactose malabsorption than the Han and Mongolian, suggesting that lactose persistence may be common in herding populations of Kyrgyz which is also nomadic as Kazakh. Although, seldom research on the prevalence of lactose malabsorption in Kyrgyz, we can infer from the study of Wang on Kazakh and the milk and dairy products are the main source of Kyrgyz diet that the incident of lactose malabsorption may not be high in them. In addition, what they drink in daily life is not necessarily milk, but fermented products such as horse milk wine. Milk has a very comprehensive nutritional value and is one of the best calcium supplements. Therefore, as the result showed in this study, the consumption of milk and dairy products should still be recommended for Kyrgyz.

Although this study did not show that exercise, light diet, and daily consumption of vegetables and fruits are associated with chronic disease prevention and control literacy, it is still recognized as a healthy lifestyle and eating habit.

### Strengths and limitations

To the best of our knowledge, this is the first time to conduct a systematic study between health literacy on chronic disease prevention and behavior and lifestyle of the Kyrgyz people, which provides inspiration for future work in related fields. A clear strength of this study is the large sample size, which allowed us to conduct a broad survey of health literacy across demographic, socioeconomic, and health-related indicators. Another strength of the study is that our questionnaire is reliable. The questionnaire was formulated according to the “Chinese Resident Health Literacy Manual” and the culture and living habits of ethnic minorities in Xinjiang.

However, our work it is based on cross-sectional data, so the causal inferences should be viewed with caution. Future intervention studies can be conducted to improve the health knowledge level and health status of patients, and appropriate measures can be taken to address the contributing factors. Secondly, the chronic diseases of the residents involved in the health literacy questionnaire were self-reported by the survey subjects, which could result in imprecision and biased results. Thirdly, because of the large number of questions in our questionnaire, some respondents may lose their patience, leading to selection bias in the results.

## Conclusion

The literacy level of chronic disease prevention among Kyrgyz residents in Kezhou has improved compared with 2018. The literacy of chronic disease prevention and treatment is an influencing factor for the healthy behavior and lifestyle of the Kyrgyz residents. Government departments and social organizations should combine the work and life characteristics of the Kyrgyz residents to carry out health education on chronic diseases in a targeted manner. At the same time, combined with national health examinations and national health literacy promotion actions, a healthy and supportive environment is created, which can improve the enthusiasm of residents and encourage residents to form healthy lifestyles and eating habits.

### Electronic Supplementary Material

Below is the link to the electronic supplementary material.

Supplementary Material 1

## Data Availability

The datasets generated and/or analysed during the current study are not publicly available because of licensed but are available from the corresponding author on reasonable request.

## Abbreviations

Kezhou: Kizilsu Kirghiz Autonomous Perfecture
