# Supplementary material for: Chronic disease prevention literacy and its influence on behavior and lifestyle: a cross-sectional study in Xinjiang, China
Source: BMC Public Health. 2023 Oct 11;23:1980. doi: 10.1186/s12889-023-16884-1 (PMC10568918; doi:10.1186/s12889-023-16884-1)
Supplement: Supplementary file 1 — Supplementary Material 1 [file 12889_2023_16884_MOESM1_ESM.doc]

**Attachments**

**Items on chronic disease prevention literacy**

D01. Which of the following is an early danger sign of cancer?

(1) Abnormal lump in the body

(2) Cough, coughing phlegm, low fever, night sweats

(3) Weight gain

(4) I don’t know

D02. How many grams of salt can adults eat per day to prevent hypertension?

(1) 2g (2) 6g (3) 9g (4) 12g (5) I don’t know

D03. Do you know which of the following are risk factors for hypertension?

(1) Overweight or obese

(2) Smoking

(3) Long-term excessive alcohol consumption

(4) Long-term high-salt diet

(5) Have a family genetic history

(6) High-fat diet

(7) Mental tension, depression

(8) Lack of exercise

(9) All of the above

(10) I don’t know

D04. Do you know which of the following are risk factors for diabetes?

(1) Overweight or obese

(2) Smoking

(3) Long-term excessive alcohol consumption

(4) High-fat diet

(5) Have a family genetic history

(6) High-sugar diet

(7) Mental tension, depression

(8) Lack of exercise

(9) All of the above

(10) I don’t know

D05. Which of the following factors are associated with hyperlipidemia?

(1) A diet with high sugar, high salt and high cholesterol

(2) Drinking and smoking

(3) Lack of exercise

(4) Obesity

(5) I don’t know

D06. How does the population with high blood lipid control their blood lipid level?

(1) Take the medicine according to the doctor’s advice

(2) Control your weight and exercise more

(3) Tune your diet

(4) Keep your mood steady

(5) I don’t know

D07. Do you know which of the following people are at high risk for chronic diseases?

(1) Overweight and central obese (Waist circumference > 85 cm for men and > 80 cm for women)

(2) Blood pressure is higher than normal (Systolic blood pressure ≥140mmHg or diastolic blood pressure ≥90mmHg)

(3) Abnormal blood lipid (Elevated total cholesterol margins or elevated triglycerides)

(4) People with impaired fasting glucose (Fasting blood glucose ranged from 6.1mmol/L to 7.0mmol/L)

(5) Family history of hypertension

(6) Long-term excessive alcohol consumption (Drink more than 25 grams of liquor daily for men and 15 grams daily for women)

(7) Chronically high dietary salt (Eat more than 6 grams of salt daily)

(8) I don’t know
